# Exosomal MicroRNAs as Mediators of Cellular Interactions Between Cancer Cells and Macrophages

**DOI:** 10.3389/fimmu.2020.01167

**Published:** 2020-06-11

**Authors:** Yoojung Kwon, Misun Kim, Youngmi Kim, Hyun Suk Jung, Dooil Jeoung

**Affiliations:** ^1^Department of Biochemistry, College of Natural Sciences, Kangwon National University, Chuncheon, South Korea; ^2^Institute of New Frontier Research, College of Medicine, Hallym University, Chuncheon, South Korea

**Keywords:** anti-cancer drugs, cancer cells, cellular interactions, exosomes, macrophages, microRNAs

## Abstract

Tumor microenvironment consists of cancer cells and various stromal cells such as endothelial cells, cancer-associated fibroblasts (CAFs), myeloid-derived suppressor cells (MDSCs), neutrophils, macrophages, and other innate and adaptive immune cells. Of these innate immune cells, macrophages are an extremely heterogeneous population, and display both pro-inflammatory and anti-inflammatory functions. While M1 macrophages (classically activated macrophages) display anti-tumoral and pro-inflammatory functions, M2 macrophages display pro-tumoral and anti-inflammatory functions. Cellular interactions and molecular factors in the tumor microenvironment affect the polarization of macrophages. We review molecules and immune cells that influence the polarization status of macrophages. Tumor-associated macrophages (TAMs) generally express M2 phenotype, and mediate many processes that include tumor initiation, angiogenesis, and metastasis. A high number of TAMs has been associated with the poor prognosis of cancers. MicroRNAs (miRNAs) have been known to regulate cellular interactions that involve cancer cells and macrophages. Tumor-derived exosomes play critical roles in inducing the M1 or M2-like polarization of macrophages. The roles of exosomal miRNAs from tumor cells in the polarization of macrophages are also discussed and the targets of these miRNAs are presented. We review the effects of exosomal miRNAs from TAMs on cancer cell invasion, growth, and anti-cancer drug resistance. The relevance of exosomal microRNAs (miRNAs) as targets for the development of anti-cancer drugs is discussed. We review recent progress in the development of miRNA therapeutics aimed at elevating or decreasing levels of miRNAs.

## Introduction

### The Tumor Microenvironment Modulates Cancer Progression

**Figure 1:**
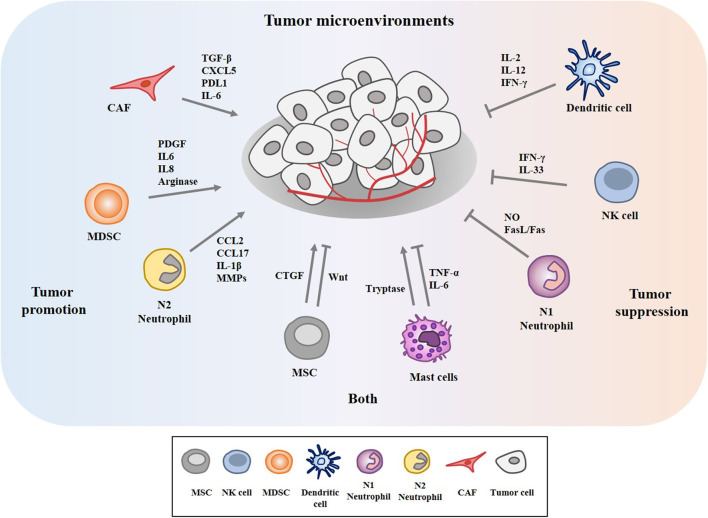
Tumor-stroma interactions in the tumor microenvironment modulate cancer progression. The tumor microenvironment comprises cancer cells, various stromal cells, and extracellular matrix. These stromal cells include endothelial cells, fibroblasts, and innate and adaptive immune cells. These cells can enhance or inhibit cancer progression by secreting cytokines, growth factors, and various other molecules. Mesenchymal stem cells (MSCs) and mast cells can enhance and inhibit cancer progression.

The tumor microenvironment (TME) is the environment surrounding tumor. The tumor microenvironment is critical for the initiation and promotion of cancer (1). The tumor microenvironment consists of various components such as endothelial cells, cancer associated fibroblasts (CAFs), innate and adaptive immune cells, and extracellular matrix. These innate immune cells include dendritic cells (DC), macrophages, neutrophils, myeloid-derived suppressor cells (MDSC), mesenchymal stem cells (MSCs), and natural killer cells (NK). CAFs release various growth factors and cytokines and mediate cancer progression (2–4). MDSCs derived by C-X-C Motif Chemokine Ligand 17 (CXCL17) enhances the metastatic potential of breast cancer cells through platelet-derived growth factor (PDGF) (5). MDSCs suppresses cytolytic T lymphocytes (CTL) activity via interleukin 6 (IL6)/IL8-arginase (Arg) axis to promote gastric cancer cell progression (6). DCs stimulated by IL37 enhance the anti-tumor effect of CD8^+^ T cells by secreting cytokines such as secrete cytokines such as IL-2, IL-12, and interferon- γ (IFN-γ) (7). Neutrophils account for up to 70% of circulating leukocytes and are the first line of defense against pathogens. Neutrophils are classified into N1 neutrophils and N2 neutrophils. N1 neutrophils are inflammatory and anti-tumorigenic and N2 neutrophils are anti-inflammatory and tumorigenic, respectively. Neutrophils can promote or inhibit tumor progression by secretion of cytokines and growth factors. Neutrophils enhance extravasation of tumor cells through the secretion of IL1β and matrix metalloproteinases (MMPs) (8). Neutrophils mediate hepatocellular carcinoma progression by C-C Motif Chemokine Ligand 2 (CCL2) and CCL17 (9). Neutrophils suppress lung cancer cell proliferation by Fas Ligand (Fas L)/Fas pathway (10). Hepatocyte growth factor (HGF)/MET proto-oncogene- dependent nitric oxide (NO) release by neutrophils suppresses tumor growth (11). MSCs mediate gastric cancer progression by connective tissue growth factor (CTGF) (12). Human umbilical cord mesenchymal stem cells suppress cholangiocarcinoma cell growth by regulating Wingless-Type MMTV Integration Site Family (Wnt)/β-catenin and phosphoinositide-3 kinase (PI3K)/Akt signaling pathway (13). NK cells suppress tumor immune evasion by IFN-γ (14). NK cells suppress the metastatic potential of cancer cells by IL-33 (15). Tryptase released by mast cells enhances tumor cell metastasis through the janus kinase (JAK)-signal transducer and activator of transcription (STAT) pathway (16). Human mast cells induce apoptosis of breast cancer cells through tumor necrosis factor-α (TNF-α) (17). Mast cell derived IL-6 suppresses proliferation of glioma cells by abolition of STAT3 signaling and the down-regulation of glycogen synthase kinase (GSK3β) (18). Figure 1 depicts tumor-stroma interactions that modulate cancer progression.

## Macrophages In the Tumor Microenvironment

The most abundant stromal cells within the tumor microenvironment are macrophages. Macrophages modulate immune responses through antigen presentation and phagocytosis, and also play important roles in tissue repair and wound healing. Depletion of peritoneal macrophages suppresses the progression of ovarian tumor (19). Macrophages are highly plastic and classified into two distinct phenotypes, M1 and M2. Depending on the type of stimuli from their milieu, macrophages show either M1 or M2 phenotype (20, 21). M1 macrophages (classical) display pro-inflammatory and M2 macrophages display anti-inflammatory function, respectively. Circulating monocytes, recruited into the tumor sites by monocyte chemoattractant 1 (MCP1), are differentiated into tumor associated macrophages (TAMs). TAMs could represent up to 50% of the tumor mass, and play a key role in tumor development (22). TAMs comprise distinct populations that share features of both M1 and M2 macrophages; however, most studies have shown that TAMs are anti-inflammatory, and correlate with a poor prognosis of cancers (23–26).

M1 macrophages are activated by cytokines such as interferon γ (IFNγ) and produce interleukin-1 (IL-1), IL-6, IL-12, tumor necrosis factor (TNF)-a, reactive oxygen species (ROS), and nitric oxide (NO) (20, 23). Pattern recognition receptors (Toll-like receptors, C-type lectins, retinoic acid inducible gene-like receptors) are necessary for the M1 macrophages polarization. These receptors recognize molecular patterns such as lipopolysaccharide (LPS), and increase the production of pro-inflammatory cytokines (IL-6, IL-12, tumor necrosis factor- α (TNF-α), etc.). Classically activated M1 macrophages are also induced by IFN-γ, alone or in combination with cytokines [e.g., TNF-α and Granulocyte Macrophage Colony-Stimulating Factor (GM-CSF)] (27). M1 macrophages can enhance tumor specific antigen presentation by increasing the expression levels of the major histocompatibility complex (MHC) class I and II molecules (28). M1 macrophages convert L-arginine into citrulline and NO by inducible nitric oxide synthase (iNOS) (28).

**Figure 2:**
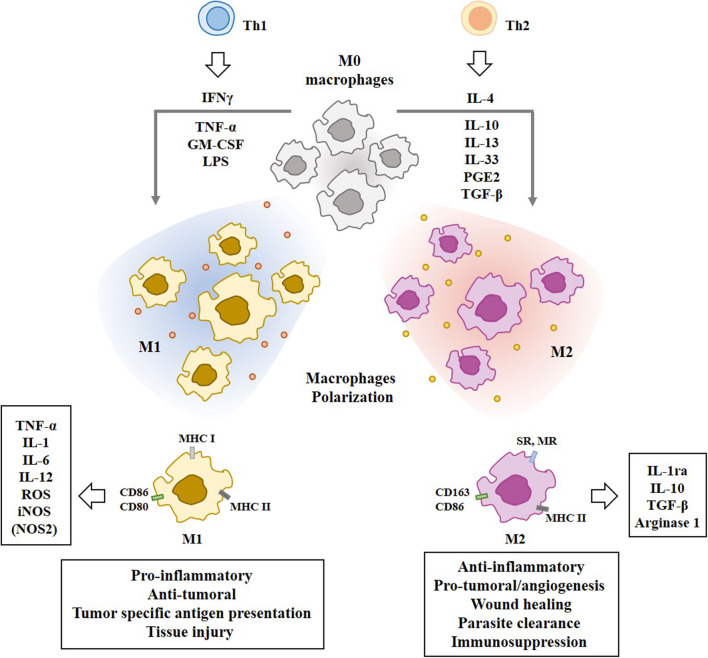
Polarization of macrophages by T-helper cells. Tumor associated macrophages (TAMs) are composed of pro-inflammatory M1 macrophages and anti-inflammatory M2 macrophages. IFN-γ, TNF-α, LPS, and GM-CSF induce M1 macrophages polarization. Th2 cytokines induce M2 macrophages polarization. Mo macrophages are circulating monocytes. M1 macrophages display higher expression level of MHC class I than M2 macrophages. M1 macrophages display higher expression level of iNOS than M2 macrophages. M2 macrophages display higher expression level of CD163 than M1 macrophages. Molecules that mediate effects of M1 macrophages or M2 macrophages are shown. SR denotes scavenger receptor. MR denotes mannose receptor. Nitric oxide synthase NOS2 (iNOS) in M1 macrophages converts L-arginine into citrulline and NO. Arginase 1 in M2 macrophages converts L-arginine into polyamine and urea.

T helper 2 (Th2) cytokines, such as IL-4, IL-10, and IL-13, prostaglandin E2 (PGE2), and transforming growth factor-β (TGF-β), induce the differentiation of monocytes into the alternative M2 macrophages which play essential roles in humoral immunity, wound healing, parasite clearance, dampening of inflammation, angiogenesis, and immunosuppression (29), and tissue remodeling (30). IL-33, a cytokine of the IL-1family, is associated with Th2 and M2 polarization (31). M2 macrophages produce and secrete IL-10 and TGF-β to inhibit M1 polarization of macrophages through the suppression of IL-12 (32). M2 macrophages convert L-arginine into polyamine and urea by Arg1 (28). Figure 2 shows the polarization of macrophages by T-helper cells and molecules that mediate various functions of the M1 and M2 macrophages.

## Cellular Interactions In the Tumor Microenvironment

The phenotype of macrophages within the tumor microenvironment is significantly affected by cellular interactions that involve cancer cells, immune cells, endothelial cells, and fibroblasts. Vascular endothelial growth factor A (VEGFA) from hepatic cancer cells (HCC) activates VEGFR2 to enhance tube formation, and the invasion potential of vascular endothelial cells (33). CC chemokine ligand 2 (CCL2)-CCR2 signaling is necessary for the activation of endothelial cells by cancer cells (34). CCL5 derived from cancer cells is necessary for tumor angiogenesis and metastasis (35). Clinical studies have revealed interactions between cancer cells (including ovarian cancer) and TAMs (36). Tumor-derived CCL2 is a monocyte-chemotactic protein and its high level correlates with high numbers of TAMs in tumor tissue and a poor prognosis (37). Tumor-derived VEGF, platelet-derived growth factor (PDGF), macrophage colony stimulating factor (M-CSF), and interleukin IL-10 leads to the recruitment of macrophages (38, 39). Renal cell carcinoma-derived IL-10 induces the M2 polarization of macrophages (40). Nicotinamide adenine dinucleotide phosphate oxidase 4 (NADPH oxidase 4) recruits M2 macrophages via the rho-associated protein kinase (ROCK)/PI3 kinase-dependent production of cytokines, thus contributing to cancer cell growth (41). Human monocytes exposed to culture medium from thyroid tumor cell lines undergo M2-like polarization of macrophages, showing high cluster of differentiation 206 (CD206) and low MHC class II markers. Thyroid tumor-derived prostaglandin E2 (PGE2) induces the M2 polarization of macrophages (42). Recombinant endothelin-1, a 21-amino acid peptide, induces the M2 macrophages polarization and leads to the increased expression levels of CD204, CD206, CD163, IL-10, and CCL22, and the production of matrix metalloproteinase-9 (MMP-9) (43).

TAMs activate tumor stem cells and enhance the metastatic potential of cancer cells (44, 45). TAMs induce the vascularization of tumor tissue by producing VEGF, PDGF, and transforming growth factor (TGF)-β (46). TAMs enhance the tumorigenic potential of cancer cells and induce anti-cancer drug resistance through cathepsin β and milk-fat globule epidermal growth factor (EGF)-VIII (47–49). TAMs produce MMPs to induce lung cancer cell invasion (50). Intraperitoneal TAMs enhance the migration and invasion of gastric cancer cells via IL-6 (51). IL-10-derived from M2 macrophages enhances the growth potential of non-small cell lung cancer cells (NSCLCs) by activating cancer stemness via janus kinase (JAK)/signal transducer and activator of transcription 1 (STAT1)/nuclear factor-kappa B (NF-kB) pathway (52). TAMs secrete TGF-β1, and promotes the proliferation and invasion potential of colorectal cancer cells (53). TAMs secrete IL-35 to promote the metastatic potential of cancer cells by activating JAK2-STAT6-GATA-bnding protein 3 (GATA3) signaling (54).

**Figure 3:**
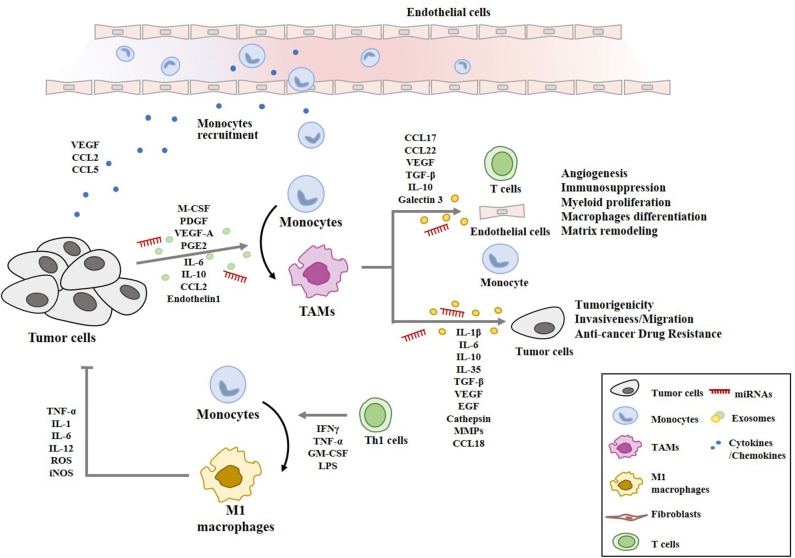
Cellular interactions involving macrophages in tumor microenvironment. Cancer cells secrete chemoattractants and growth factors (CCL2, CCL5, VEGF, M-CSF) to recruit blood monocytes. Tumor-derived M-CSF, PDGF, IL-6, IL-10, TGF-β, VEGF-A, CCL2, PGE2, and endothelin 1 induce the polarization into M2-like macrophages with pro-tumor functions (TAMs). TAMs-derived cytokines/growth factors (IL-1β, IL-6, IL-10, IL-35, TGF-β, VEGF, and EGF), cathepsin, and MMPs enhance tumor cell proliferation, invasion, anti-cancer drug-resistance, and remodeling of extracellular matrix. TAMs-derived chemokines (CCL17, CCL18, and CCL22) recruit naïve and Th2 lymphocytes, which results in ineffective anti-tumor immune response. miRNAs denote microRNAs. M1 macrophages are activated by Th1 cells. M1 macrophages display anti-tumoral functions through TNF-α, IL-1, IL-6, IL-12, ROS, and iNOS.

Myeloid cells infiltrating into tumor stroma mostly express M2 markers play essential roles in immunosuppression, tumor angiogenesis, and tumor metastasis (23). IL-10, released by TAMs, increases the number of regulatory T (Treg) cells by activating Forkhead Box P3 (Foxp3) (55). TAMs-derived TGFβ induces Treg response by cellular interaction (56). TAMs induce immunosuppressive and tumorigenic functions, and express the enzyme Arg1. Anti-programmed cell death ligand 1 (PDL1) therapy decreases the number of TAMs with Arg1^+^ to suppress cancer progression (57). TAMs suppress CD8^+^T cell activity by releasing CCL17, CCL22, TGF-β, IL-10, Arg1, and galectin-3 (58). Genetic ablation of colony stimulating factor (CSF1) in colorectal cancer cells inhibits the influx of immunosuppressive CSF1R^+^ TAMs in tumors. This reduction results in CD8^+^ T cell attack on tumors, but a compensatory increase in Foxp3^+^ Treg cells limits its effect on tumor growth (59). Cancer cells and TAMs induce CD4^+^FoxP3- type I regulatory T cells (Tr1) cells in glioblastoma (GBM) patients. Co-culture of Tr1 with CD8^+^ T cells reduces the tumor-specific cytotoxic effects of CD8^+^T cells (60). TAMs-derived TGF-β induces the macrophage polarization of M1 into M2-like phenotype via the upregulation of SNAIL, and blockade of TGF-β/SNAIL signaling restores the production of pro-inflammatory cytokines (61). Figure 3 provides an overview of the cellular interactions involving cancer cells and macrophages within the tumor microenvironment.

## Tumor-Derived Exosomal miRNAs Regulate the Polarization of Macrophages

In the tumor microenvironment, cancer cells secrete exosomes. Exosomes are lipid bilayer membrane vesicles of (50–100) nm that are derived from the luminal membrane of multivesicular bodies, which are constitutively released by fusion with the cell membrane (62). Most eukaryotes release exosomes to various biological fluids such as blood, lymph, and milk. Exosomes are particularly enriched in various tumor microenvironment (63). Cancer cells generate more exosomes than normal cells, and circulating exosomes levels are increased in the blood of cancer patients when compared to healthy individuals (64). Exosomes can affect the phenotype of recipient cells as it can transfer their contents to recipient cells. Tumor-derived exosomes enhance tumorigenic potential, and metastatic potential by increasing the expression of MET oncogene in bone marrow progenitor cells from individuals with melanoma (65). Immunosuppressive exosomes of tumor can enhance tumor growth by inducing apoptosis of cytotoxic T cells (66). Exosomes from EGFR2-overexpressing anti-cancer drug resistant breast cancer cells contain immunosuppressive cytokine such as TGF-β and confers anti-cancer drug resistance in anti-cancer drug sensitive breast cancer cells (67).

miRNAs represent one of the predominant RNAs contained in exosomes (68, 69). miRNAs are short, non-coding RNAs that regulate the expression of complementary mRNAs (70). miRNAs function in RNA silencing and post-transcriptional regulation of gene expression (71). miRNAs are initially transcribed as long primary miRNAs (pre-miRNAs), and are subsequently processed by the microprocessor complex that contains the double strand RNA-specific endoribonuclease DROSHA and its RNA binding partner DiGeorge Syndrome critical region gene 8 (DGCR8) (72). Mature miRNAs bind to multiple targets (73). miRNAs are frequently deregulated in many types of cancer and regulate tumor progression, metastasis, and anti-cancer drug resistance (74–76). miRNAs paly critical roles in tumor immunity by regulating the polarization of macrophages (77).

Exosomes protect miRNAs from degradation, enabling them to be stably expressed in the extracellular space (78). Exosomal miRNAs derived from cancers act as mediators of interactions between cancer cells and the tumor microenvironment (79, 80). The exosomal miRNAs mediate communication between colon cancer cells and TAMs and reprograms macrophages to create a microenvironment for tumor growth; this microenvironment enables cancer cells to metastasize (81). Modification or inhibition of exosomal miRNAs might offer a valuable therapeutic strategy in cancer.

Tumor-derived exosomes (TDEs) deliver proteins, messenger RNAs (mRNAs), and miRNAs to various stromal cells in the tumor microenvironment. Epithelial ovarian cancer (EOC)-derived exosomal miR-222 activates STAT3 signaling and induces the M2 polarization of macrophages (82). Exosomal miRNAs (miR-21–3p, miR-125 b-5p, and miR-181 d-5p) induce the M2 polarization of macrophages (83). These miRNAs are induced by hypoxia via hypoxia-inducible factors (HIFs) (83). Exosomal miR-940 derived from EOC cells stimulates the M2 polarization of macrophages (84). Hypoxic conditions induce the expression of exosomal miR-940 in EOC cells (84). Exosomal miR-222 from EOC cells induces the M2 polarization of macrophages by targeting the suppressor of cytokine signaling 3 (SOCS3) (82, 85). Exosomal miRNAs (miR-25-3p, miR-130b-3p, miR-425-5p), upregulated in colorectal cancer cells by activation of the CXCL12/CXCR4 axis, induce the M2 polarization of macrophages by regulating the expression of phosphatase and tensin homolog (PTEN) through the activation of phosphatidyl inositol 3 kinase (PI3K)/Akt signaling pathway (74). Exosomal mR-203 from colorectal cancer cells induces the M2 polarization of macrophages and enhances the metastatic potential of colorectal cancer cells by increasing the expression levels of CD163 and STAT3 (86). Exosomal miR-145 from colorectal cancer cells induces the M2 polarization of macrophages by decreasing the expression of histone deacetylase 11 (HDAC11) (87). P53 is responsible for the increased expression of miR-145 (87). Exosomal miR-150 from hepatic cancer cells increases the secretion of VEGF from M2 macrophages (88). Exosomal miR-146a from hepatic cancer cells induces the M2 polarization of macrophages and suppresses the anti-tumor function of T cells by increasing the expression of programmed death-1 (PD-1) in T cells (89). Spalt Like Transcription Factor 4 (SALL-4) binds to the promoter sequences of miR-146a, and directly increases the expression of miR-146a (89). Endoplasmic reticulum (ER)-stressed hepatic cancer cells release exosomes to upregulate programmed death-ligand 1 (PD-L1) expression in macrophages, which subsequently inhibits anti-tumor T-cell function through an exosome miR-23a-3p-PTEN-AKT pathway (90). Exosomal miR-301a-3p from pancreatic cancer cells induces the M2 polarization of macrophages by targeting PTEN/PI3Kγ pathway (91). HIF-1a or HIF-2a is responsible for the increased expression level of exosomal miR-301a-3p (91).

**Figure 4:**
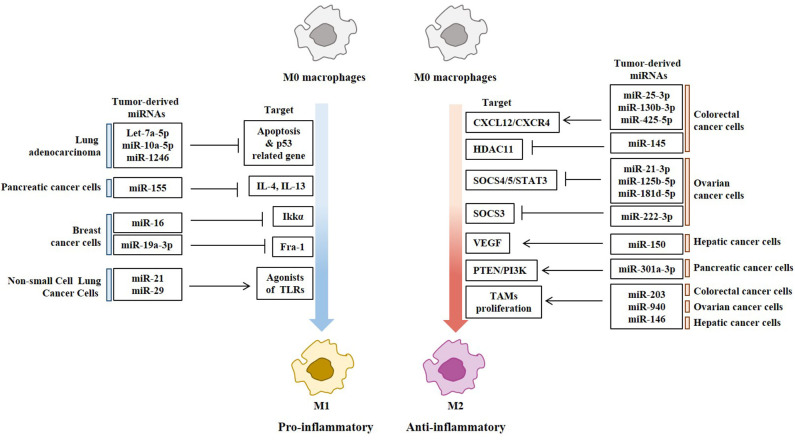
Tumor-derived miRNAs regulate polarization of macrophages. Tumor-derived miRNAs induce polarization of anti-tumoral and pro-inflammatory M1 macrophages or Pro-tumoral and anti-inflammatory M2 macrophages.

Exosomes of miR-125b-transsfectd lung cancer cells display reprogrammed miRNA expression profiles and the miRNAs in the exosomes regulate expression levels of apoptosis-related genes (92). Exosomal miR-19a-3p suppresses breast cancer progression by inducing M1 macrophages polarization by regulating the expression levels of Fos-related antigen 1 (Fra-1), VEGF, and signal transducer and activator of transcription 3 (STAT3) (93). Exosomal miR-21 and miR-29 from NSCLCs bind to toll-like receptors (TLRs), and induce the M1 polarization of macrophages (94). Epigallocatechin-3-gallate (EGCG) has anti-tumor and anti-inflammatory activities. Exosomes from EGCG-treated 4T1 (breast cancer) cells inhibit the infiltration of TAMs and differentiation into M2 macrophages. miR-16 expression is increased in both cancer cells and exosomes by ECGC treatment. miR-16 decreases I kappa B kinase α (IKKα) expression in TAMs, and subsequently inhibits NF-κB pathway, which is critical for TAMs infiltration and the M2 polarization of macrophages (95). Exosomal miR-155 and miR-125b-5p derived from pancreatic cancer cells induce repolarization of M2 macrophages into M1 macrophages by increasing expression levels of iNOS and TNF-α (96). Figure 4 shows the tumor-derived miRNAs that regulate the polarization of macrophages. Table 1 shows the targets of these miRNAs. Table 2 shows the chromosomal localizations of these miRNAs and factors regulating the expression levels of these miRNAs. Some of these miRNAs form cluster. The expression of clustered miRNAs is believed to be co-regulated and these miRNAs display similar expression patterns (97, 98). These miRNAs may be involved in common biological processes.

**Table 1:** Summary of tumor-derived miRNAs with known functions in polarization of macrophages.

**miRNAs**	**Targets**	**miR-Transfer**	**Donor cell**	**Macrophage polarization**	**Reference**
miR-16	NF-kB pathway	Exosomal	Breast cancer cells	Classically activated macrophages(M1)	(95)
miR-21	TLRs	Exosomal	NSCLC		(94)
miR-29	TLRs	Exosomal	NSCLC		(94)
miR-155	IL-4, IL-13	Exosomal	Pancreatic cancer cells		(96)
miR-21-3p	SOCS4/5/STAT3	Exosomal	Ovarian cancer cells	Alternatively activated macrophages(M2)	(83)
miR-25-3p	PTEN/PI3K/Akt	Exosomal	Colorectal cancer cells		(74)
miR-125b-5p	SOCS4/5/STAT3	Exosomal	Ovarian cancer cells		(83)
miR-130b-3p	PTEN/PI3K/Akt	Exosomal	Colorectal cancer cells		(74)
miR-145	HDAC11	Exosomal	Colorectal cancer cells		(87)
miR-146	TAMs	Exosomal	Hepatic cancer cells		(89)
miR-150	VEGF	Exosomal	Hepatic cancer cells		(88)
miR-181d-5p	SOCS4/5/STAT3	Exosomal	Ovarian cancer cells		(83)
miR-203	TAMs	Exosomal	Colorectal cancer cells		(86)
miR-222-3p	SOCS3	Exosomal	Ovarian cancer cells		(82)
miR-301a-3p	PTEN/PI3K	Exosomal	Pancreatic cancer cells		(91)
miR-425-5p	PTEN/PI3K/Akt	Exosomal	Colorectal cancer cells		(74)
miR-940	TAMs	Exosomal	Ovarian cancer cells		(84)

*Sources of these miRNAs and their targets are described*.

**Table 2:** Summary of chromosomal localization and expression regulation of exosomal miRNAs.

**miRNAs**	**miRNAs species**	**miRNAs Genomic location**	**Clustered miRNAs**	**Models**	**Regulation of miRNAs**	**Reference**
miR-16	Mouse	Chr.3	miR-16	Breast cancer cells		(95)
miR-21	Human	Chr.17q23.1	miR-21, miR-104	NSCLC		(94)
miR-29	Human	Chr.7q32.3	miR-29a/b	NSCLC		(94)
miR-155	Human	Chr.21q21.3	miR-155	Pancreatic cancer cells		(96)
miR-21-3p	Human	Chr.17q23.1	miR-21, miR-104	Ovarian cancer cells	HIF-1α/HIF-2α	(83)
miR-25-3p	Human	Chr.7q22.1	miR-25, miR106b, miR-93	Colorectal cancer cells	CXCR4 activation	(74)
miR-125b-5p	Human	Chr.11q24.1	miR-125b, lin-4	Ovarian cancer cells	HIF-1α/HIF-2α	(83)
miR-130b-3p	Human	Chr.22q11.21	miR-130b, miR-301b	Colorectal cancer cells	CXCR4 activation	(74)
miR-145	Human	Chr.5q32	miR-143, miR-145	Colorectal cancer cells	p53	(87)
miR-146	Mouse	Chr.11	miR-146	Hepatic cancer cells	SALL4	(89)
miR-150	Human	Chr.19q13.33	miR-150	Hepatic cancer cells		(88)
miR-181d-5p	Human	Chr.19p13.12	miR-181c/d	Ovarian cancer cells	HIF-1α/HIF-2α	(83)
miR-203	Human	Chr.14q32.33	miR-203a/b	Colorectal cancer cells		(86)
miR-222-3p	Human	Chr.Xp11.3	miR-221, miR-222	Ovarian cancer cells		(82)
miR-301a-3p	Human	Chr.17q22	miR-301a	Pancreatic cancer cells	HIF-1α/HIF-2α	(91)
miR-425-5p	Human	Chr.3p21.31	miR-191, miR-425	Colorectal cancer cells	CXCR4 activation	(74)
miR-940	Human	Chr.16p13.3	miR-940, miR-3677, miR-4717	Ovarian cancer cells	hypoxic conditions	(84)

## Exosomal miRNAs of Cancer Cells Regulate Anti-Cancer Drug Resistance

Accumulating evidence suggests that the tumor microenvironment plays a pivotal role in the development of anti-cancer drug resistance (99). Exosomes from cancer cells, cancer associated fibroblasts, or immune cells, carrying miRNAs have been shown to confer anti-cancer drug-resistance. Exosomal drug-efflux pumps and miRNAs can regulate anti-cancer drug resistance (100). miR-365a-3p enhance the migration and invasion of lung cancer cells by inhibiting ubiquitin specific peptidase 33 (USP33)/ slit guidance ligand 2 (SLIT2)/ roundabout guidance Receptor 1 (ROBO1) signaling pathway (101). Exosomal miR-365 from imatinib resistant chronic myeloid leukemia cells can confer drug resistance phenotypes in imatinib-sensitive chronic myeloid leukemia cells by decreasing apoptosis (102). Exosomes from anti-cancer drug-resistant breast cancer cells increase the levels of TGFβ1 and the lymphocyte activation inhibitor PD-L1 to confer resistance to anti-cancer drugs, such as trastuzumab (103). The down-regulation of miR-155 reduces the malignancy of chordoma cells, and enhances sensitivity to anti-cancer drugs by increasing the expression of PTEN and activating PI3K-Akt-mammalian target of rapamycin (mTOR) signaling (104). miR-155 overexpression suppresses suppressor oy cytokine signaling 1 (SOCS1) expression and induces the progression of Anaplastic thyroid cancer (ATC) (105). The inhibition of SOCS1 reverses the effects of mR-155 on cancer cell proliferation (105). Exosomal miR-155 of hepatocellular carcinoma cells (HCC) binds to the 3′-untranslated region (UTR) of PTEN, and stimulates the proliferation of HCC (106).

## Exosomal miRNAs of Tams Regulate Tumor Growth and Anti-Cancer Drug Resistance

TAMs modulate various factors in the tumor microenvironment to facilitate tumor progression. TAMs secrete pro-tumorigenic factors that induce the gemcitabine resistance of pancreatic ductal adenocarcinoma (PDAC) cells (107). TAMs infiltrate solid tumors, and stimulate cell proliferation and angiogenesis (108, 109). TAMs mediate lung tumor progression enhanced by protein arginine methyl transferase 6 (PRMT6) (110). Exosomes derived from TAMs enhance the tumor growth and metastasis in mice (111). TAMs-derived exosomal miR-501-3p inhibits tumor suppressor transforming growth factor beta receptor 3 (TGFBR3) gene, and facilitates the development of PDAC (111). Exosomes derived from TAMs confer resistance to cisplatin in gastric cancer cells by activating PI3K/AKT signaling pathway (112). TAMs-derived exosomes (MDE) enhance the migration and invasion potential of colon cancer cells. In this, exosomal miR-21-5p and miR-155-5p bind to transcriptional activator brahma related gene 1(BRG1) and decreases the expression of BRG1 (113). BRG1 is a negative regulator of colorectal cancer metastasis. That study also showed that culture medium of M2 macrophages enhanced the motility, invasion, and metastasis of colorectal cancer cells. BRG1 mutation or lack of expression occurs in primary lung tumors and several cancers (114). Exosomal miR-223 from IL4-activated macrophages enhances the invasion potential of breast cancer cells by targeting myocyte enhancer factor 2 (MEF2)-β-catenin pathway (115). Overexpression of miR-223-3p enhances cell proliferation and metastasis by regulating the expression of solute carrier family 4 (SLC4A4) (116). miR-223 is upregulated in colon cancer and enhances colon cancer cell invasion and metastasis by decreasing the expression of p120 (117). Exosomal miR-223 derived from TAMs induces anti-cancer drug resistance in EOC cells by activating PTEN-PI3K/AKT pathway (118). miR-223 induces anti-cancer drug resistance in gastric cancer cells by regulating the expression of F-box and WD repeat domain-containing 7 (FBXW7) (119, 120). Adoptive transfer of miR-365 in TAMs confers resistance to gemcitabine in PDAC-bearing mice by upregulation of the triphospho-nucleotide pool in cancer cells (121). Cisplatin-stimulated TAMs enhance ovarian cancer cell migration by increasing the expression of CCL20 (122). Exosomes from plasma of EOC patients display higher expression level of miR-21, as compared to that of healthy women (123). The exosomal miR-21 confers resistance to cisplatin through the downregulation of PTEN, leading to the activation of PI3K/AKT pathway. The lack of miR-21 expression in TAMs results in anti-tumoral immune response involving the improvement of cytotoxic T-cell responses by macrophages through the induction of IL-12 and C-X-C motif chemokine 10 (124). miR-21-3p promotes proliferation and anti-apoptosis in esophageal squamous cell carcinoma (ESCC) by regulating tnf receptor associated factor 4 (TRAF4) (125). KRAS upregulates miR-21 and enhances cell migration/invasion by inhibiting tumor suppressor genes, such as neurofibromin 1 (NF1), ras p21 protein activator (RASA1), and Ras Association Domain-Containing Protein 8 (RASSF8) (126).

Exosomal miR-7 from TAMs suppresses the metastasis of EOC cells by inhibiting epidermal growth factor receptor (EGFR)/Akt/extracellular regulated kinase (ERK) pathway (127). Exosomal miR-142-3p from macrophages decreases the expression levels of stathmin-1 and insulin-like growth factor-1 receptor to inhibit the proliferation of HCCs (80).

**Figure 5:**
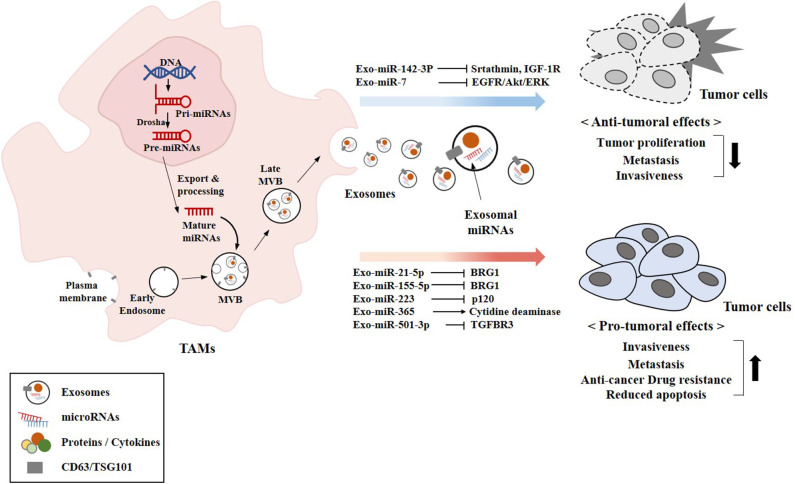
Exosomal miRNAs from TAMs exert pro-tumoral and anti-tumoral effects. The biogenesis of exosomes involves four different steps: (a) the membrane invagination; (b) endosome formation; (c) inward budding of endosomes to form multivesicular bodies (MVBs); and (d) the fusion of MVBs with the plasma membrane release the ILVs in the extracellular space by exocytosis and become exosomes. Exosomal miRNAs (miR-7 and miR-142-3p) that exert anti-tumoral effects are shown. Exosomal miRNAs (miR-21-5p and other miRNAs) that exert pro-tumoral effects are also shown. CD63 and TSG101 are surface markers of exosomes.

Figure 5 provides an overview of exosomal miRNAs from TAMs that exert tumor or anti-tumoral functions.

## Current Progress In miRNA Therapeutics

miRNAs can serve as targets for the development of anti-cancer drugs based on their roles in tumorigenesis, cancer progression, and anti-cancer drug resistance. Resistance to standard anti-cancer drugs, such as Taxol, Doxorubicin, and Cisplatin, involves the deregulated expression of miRNAs in breast cancer (128). These miRNAs can also be employed as targets for the development of anti-cancer therapeutics. The close relationship between miRNA, tumor microenvironment, and the hallmarks of cancer makes miRNAs valuable targets for the development of therapeutic strategy to complement current cancer therapies. Small interfering RNA (siRNA), was granted FDA approval in 2018. However, miRNA drugs have not yet been granted FDA approval.

miRNA-based therapeutics that decrease the levels of oncogenic miRNAs (oncomiRs) or increase the levels of tumor suppressor miRNAs have great potential as cancer therapeutics. Clinical trials of miRNA-based drugs have shown promising results for the treatment of cancer (129). Since miRNAs have multiple targets, miRNA drugs might induce unwanted side effects. Systemic delivery methods of miRNA drugs involve injection or intravenous administration. Intratumoral injections of miRNA drugs can enhance target specificity and efficacy, and minimize side effects (130, 131). Intratumoral injection of cationic liposome/pVAX-miR-143 complex (CL-pVAX-miR-143) inhibits subcutaneous tumor growth and systemic injection inhibits tumor metastasis in a dose-dependent manner in early-stage experimental lung cancer metastasis models (132).

Clinical phase 1 trial employing “TargomiR” shows promising results in patients with malignant pleural mesothelioma or non-small cell lung cancer. TargomiR delivery vehicles comprise miRNA mimic, minicells, and a targeting moiety (a specific antibody that recognizes a target protein). MesomiR-1, the targomiR drug, comprises the miRNA mimic corresponding to tumor-suppressor miR-16 and an antibody to the EGFR (133, 134).

An antagomiR is a synthetic single-helix nucleic acid, consisting of DNA or analogs, peptide or locked nucleic acids (PNAs or LNAs, respectively), or miRNA sponges, perfectly complementary to a specific miRNA target. AntagomiRs can inhibit many different mRNAs. Many miRNA drugs are in the form of antagomiRs. Many approaches are now being employed to decrease the expression levels of miRNAs that display oncogenic potential (OncomiRs). For example, CRISPR/Cas-9 system is employed to decrease the expression levels of these oncomiRs (135). Putative miRNA drugs have exhibited significant efficacy in some cancers (miR-16 in lung cancer and miR-155 in T cell lymphoma), hepatitis C (miR-122), heart abnormalities (miR-92), and pathologic fibrosis (miR-29). Phase 1 clinical trial of MRG 110, a locked nucleic acid (LNA)-modified antisense oligonucleotide to inhibit the function of miR-92, is underway. Cobomarsen (MRG-106), a locked nucleic acid-modified oligonucleotide inhibitor of miR-155, inhibits cell proliferation and activates apoptosis in human lymphotropic virus type 1 (HTLV-1+) cutaneous T-cell lymphoma (CTCL) cell lines (136). A first-in-human phase 1 clinical trial of cobomarsen in patients with CTCL is currently underway. Identification of targets of miR-155 will be necessary for the rational design of cancer therapeutics. miR-10b is up-regulated in high-grade, and significantly down-regulated in low-grade gliomas (137). miR-10b targeting drug will be tried in patients with glioblastoma multiforme with a median survival of approximately 14.6 months.

## Future Prospect

Cellular interactions within the microenvironment lead to tumor growth and progression. Understanding of cellular interactions within the tumor microenvironment is necessary for the development of anti-cancer therapeutics. TAMs play critical roles in tumorigenesis, tumor metastasis, and anti-cancer drug resistance. TAMs can therefore serve as targets for the development of anti-cancer therapeutics. Changing immunosuppressive TAMs into M1 macrophages can be used in combination with current anti-cancer immunotherapies (138). Specific deletion of TAMs might also be used in combination with current anti-cancer therapy. Inhibition of the infiltration of monocytes into the tumor site can be an effective anti-cancer therapy. Many reports suggest that circulating miRNAs can be employed as diagnostic markers for cancers (139). Identification of the signature miRNAs of each cancer will be helpful for the diagnosis of cancers, and the development of anti-cancer therapeutics. Identification of the target genes and their implicated functions, along with an *in vitro* and *in vivo* preclinical research models, has made it possible for the development of anti-cancer drugs employing miRNAs. miRNAs that induce the M2 polarization of macrophages can serve as targets for the development of anti-cancer drugs. Macrophages miRNAs that regulate ant-cancer drug-resistance, tumor growth, and tumor metastasis can also serve as targets for the development of anti-cancer drugs. miRNA therapeutics offer a valuable approach for the development of anti-cancer therapeutics.

## Author Contributions

DJ wrote the manuscript. YKw and MK made the figures and tables. HJ helped in editing. YKi and HJ provided intellectual output in the manuscript.

## Conflict of Interest

The authors declare that the research was conducted in the absence of any commercial or financial relationships that could be construed as a potential conflict of interest.
